# Atypical Stress Cardiomyopathy: A Case Report

**DOI:** 10.7759/cureus.27786

**Published:** 2022-08-08

**Authors:** Kameron Tavakolian, Mihir Odak, Brett Miller, Anton Mararenko, Savannah Nightingale, Steven Douedi, Swapnil V Patel

**Affiliations:** 1 Internal Medicine, Jersey Shore University Medical Center, Neptune City, USA; 2 Obstetrics and Gynecology, Monmouth Medical Center, Long Branch, USA

**Keywords:** cardiomyopathy, chest pain, midventricular hypokenesis, takotsubo cardiomyopathy, atypical stress cardiomyopathy

## Abstract

Stress cardiomyopathy is a reversible cause of cardiomyopathy characterized by a transient dysfunction in left ventricular systolic function. It is most common in postmenopausal women and usually occurs following an emotional and/or physical stressor. The classical imaging finding is described as left ventricular apical ballooning. However, several rare variants have been reported with a strikingly different regional distribution of wall motion abnormalities. We describe a case of a 65-year-old female who was found to have stress cardiomyopathy with variant wall motion abnormality on the left ventriculogram without a preceding stressor event. We postulate that there may be a link between stress-induced cardiomyopathy without a preceding stressor event and variant wall motion abnormality patterns.

## Introduction

Stress cardiomyopathy is a rare and reversible cause of cardiomyopathy that is characterized by transient left ventricular systolic dysfunction closely mimicking myocardial infarction in the absence of acute plaque rupture on angiography. First described in Japan in 1990, it is classically referred to as Takotsubo cardiomyopathy for its close resemblance to a Japanese octopus trap and appears on left ventricular ventriculography [1]. Classically, stress cardiomyopathy is seen in postmenopausal women with one registry-based study of 1,750 patients reporting 90% of women with a mean age of 67 years old [2]. As the name suggests, emotional and/or physical stress are usually the instigating trigger, although up to 29% of patients may have no predisposing stressor [2]. 

Characteristic left ventricular apical ballooning is the hallmark feature of stress cardiomyopathy. However, there are several other rare variants with markedly different wall motion abnormalities well documented. We describe an atypical case of stress cardiomyopathy in a patient with no apparent trigger and nonclassical wall motion abnormalities seen on left ventriculography. 

## Case presentation

**Figure 1:**
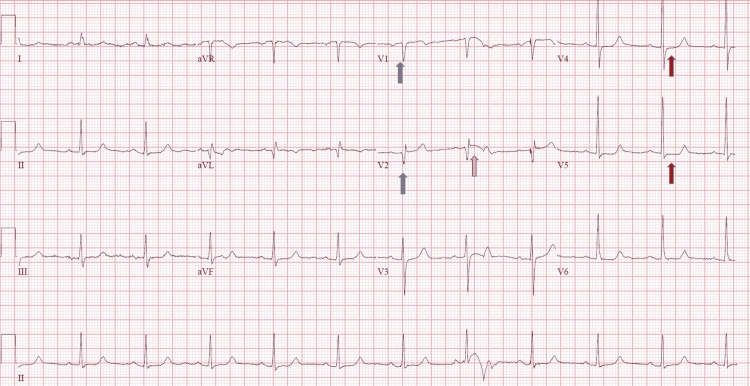
Electrocardiogram (ECG) conducted on admission The ECG shows Q waves in leads V1-V2 (grey arrows) with 1 mm ST-segment elevations in V2 (pink arrow), and 1 mm ST-segment depressions in the lateral precordial leads (red arrows).

A 65-year-old-female with a past medical history significant for hyperlipidemia and hypothyroidism presented to our hospital complaining of sudden onset chest pain for two hours. The chest pain was described as eight out of 10, pressure-like, substernal, non-radiating, and there were no aggravating or relieving factors. Her pain was associated with shortness of breath, diaphoresis, and anxiety. She denied any fever, chills, nausea, vomiting, lower extremity swelling or pain, previous episodes of chest pain, and any recent physical/emotional stress. Vital signs on presentation were within normal limits. Laboratory studies were significant for troponin of 0.02 ng/mL (reference range: <0.04ng/mL) and leukocytosis with a white blood cell count of 15.0 x10^3^/uL (reference range: 4.5 - 11.0 (x10^3^/uL). An electrocardiogram (ECG) demonstrated Q waves in leads V1-V2 with 1 mm ST-segment elevations in V2 and 1 mm ST-segment depressions in the lateral precordial leads (Figure 1). A bedside echocardiogram showed septal wall motion hypokinesis. The patient was given 325 mg aspirin and 0.4 mg sublingual nitroglycerin. Intravenous nitroglycerin infusion at a rate of 2 mcg/kg/min was temporarily initiated and had to be discontinued due to hypotension. Despite these interventions, the patient’s chest pain persisted.

**Figure 2:**
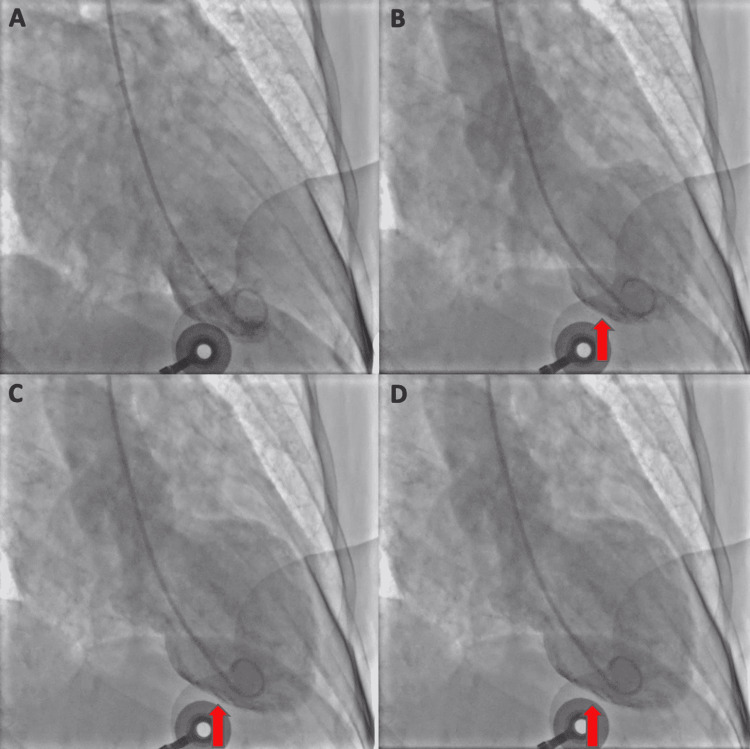
Left ventriculogram Left ventriculogram demonstrating diastolic (A) and systolic (B, C, and D) phases of the cardiac cycle with hypokinesis of the mid-distal anterior wall and mid-inferior wall (red arrows) on the right anterior oblique view.

The patient was taken emergently for a left heart catheterization for fear of acute coronary syndrome, which showed nonobstructive coronary artery disease with a mid-lateral circumflex lesion of 35% stenosis. The left ventriculogram demonstrated hypokinesis of the mid-distal anterior wall and the mid-inferior wall on the right anterior oblique view (Figure 2). Due to these findings, a formal transthoracic echocardiogram was done showing a left ventricular ejection fraction of 41% to 45% and global hypokinesis of the mid to apical walls with preserved hypercontractile basal wall motion consistent with stress cardiomyopathy. After catheterization, troponins trended and peaked at 0.64 ng/mL, likely due to type II myocardial infarction. On day two of the patient’s admission, her chest pain resolved, and she was started on metoprolol tartrate 25 mg twice a day. Angiotensin-converting enzyme inhibitor/angiotensin receptor block initiation was limited by borderline hypotension and was to be reevaluated on an outpatient basis. After one more day of observation, the patient was medically stable for discharge with a follow-up scheduled with our cardiology team for a repeat echocardiogram. At the six-month follow-up, the patient was found to have a left ventricular ejection fraction >55%. 

## Discussion

Stress cardiomyopathy is an acute and reversible cause of left ventricular dysfunction that is often preceded by a stressful event [3]. Although not fully understood, it is believed that the pathophysiology of the disorder involves catecholamine excess, microvascular dysfunction, and coronary artery vasospasm [4]. Prevalence is estimated to be 2% of all patients presenting with clinical manifestation of acute coronary syndrome [5]. Clinical presentation is similar to that of acute coronary syndrome, with the most common presenting symptoms being chest pain, dyspnea, and syncope [2].

Diagnostic work-up for stress cardiomyopathy usually involves electrocardiogram, cardiac biomarkers, coronary angiography, and echocardiography or ventriculography [3]. The electrocardiogram is abnormal in greater than 95% of patients and usually shows signs of ischemia [6]. One review by Templin et al. analyzed 1,750 patients in The International Takotsubo Registry and reported elevated troponin in 87%, elevated brain natriuretic peptide in 82.9%, ST-segment elevation in 43.7%, and reduced left ventricular ejection fraction (LVEF) in 86.5% of patients [2]. Our patient presented with normal troponin level, ST-segment elevations in leads V1 and V2, and reduced LVEF. To diagnose stress cardiomyopathy, obstructive coronary artery disease and/or acute plaque rupture must be excluded. However, Templin et al. report that 15.3% of patients had coexisting coronary artery disease. In these patients, the diagnosis can still be made if wall motion abnormalities occur in a distinct location. Coronary angiography in our patient revealed mild, nonobstructing coronary artery disease in the left circumflex artery.

Five characteristic wall motion abnormalities seen on echocardiogram or ventriculography have been described in stress cardiomyopathy. Templin et al. identified apical type in 81.7%, midventricular type in 14.6%, basal type in 2.2%, and focal type in 1.5% of patients. A fifth type characterized by global hypokinesis has also been described [7]. Our patient exhibited the midventricular type which is characterized by midventricular hypokinesis with relative sparing of the apex. 

Stress cardiomyopathy is generally managed with supportive therapy and carries an excellent prognosis with a low risk of recurrence. However, some patients may develop signs of cardiogenic shock. In these patients, an echocardiogram plays a crucial role in directing therapeutic management. The prevalence of left ventricular outflow tract obstruction (LVOTO) in stress cardiomyopathy is approximately 12.8% [8] and must be considered before treatment initiation. Furthermore, the presence of LVOTO is associated with a worse prognosis and is seen more often in patients with major cardiac events such as acute heart failure, cardiogenic shock, and cardiac death [8]. Patients without LVOTO are generally treated with positive inotropic agents, vasopressors, and mechanical circulatory support with short-term follow-up assessment to monitor for the development of LVOTO [3]. Patients with LVOTO may benefit from beta-blockers and pure alpha agonists as these interventions may reduce LVOTO and improve cardiac output [3]. 

## Conclusions

We report a unique case of stress cardiomyopathy in a patient without an emotional or physical stressor and an atypical wall motion abnormality pattern. Among cases of stress cardiomyopathy, very few present without a physical or emotional trigger, and even fewer exhibit midventricular hypokinesis. We postulate that there may be a link between stress-induced cardiomyopathy without a preceding stressor event and variant wall motion abnormality patterns. Further research is warranted to determine whether a statistically significant difference in outcomes exists amongst this patient population.
